# Microbial‐Immune Interplay in CNS Autoimmune Diseases: Lessons from Animal Models and Clinical Studies

**DOI:** 10.1002/eji.70135

**Published:** 2026-02-02

**Authors:** Matteo Ceccon, Francesca Ronchi

**Affiliations:** ^1^ Institute of Microbiology, Infectious Disease, and Immunology Charité – Universitätsmedizin ‐ Berlin Berlin Germany

**Keywords:** CNS autoimmune diseases, immune system, microbiota, mouse models, multiple sclerosis

## Abstract

The gut microbiota plays a key role in shaping and educating host immunity, and it may also influence the central nervous system (CNS). Changes in the composition and function of commensal microbes can trigger inflammation and abnormal immune activation, contributing to disorders such as multiple sclerosis (MS), neuromyelitis optica spectrum disorder (NMOSD), myelin oligodendrocyte glycoprotein antibody‐associated disease (MOGAD), autoimmune encephalitis (AIE), neuropsychiatric lupus (NPSLE), and narcolepsy. The microbiota has been linked to disease risk or protection via the production of its metabolites, products, or antigens that could modulate host immune responses, consequently causing CNS inflammation. This review highlights patient studies investigating the mechanisms through which the human microbiota is involved in CNS autoimmunity by modulating the host immune system. Future research should focus on defining causal relationships, elucidating molecular mechanisms, and addressing the different members of the intestinal microbiota to translate microbiota modulation into clinical interventions for CNS autoimmune diseases.

## Graphical abstract

**Mechanism of action of the intestinal microbiota on CNS autoimmune diseases**. Environmental factors shape gut microbiota composition; dysbiosis alters microbial metabolites and antigenic signals. These modulate innate and adaptive immunity, affect barrier integrity, and influence CNS‐resident cells such as microglia and astrocytes, contributing to immune‐mediated CNS pathology. Multiple sclerosis (MS), neuromyelitis optica spectrum disorder (NMOSD), myelin oligodendrocyte glycoprotein antibody‐associated disease (MOGAD), autoimmune encephalitis, and neuropsychiatric lupus (NPSLE). The image provided is free of copyright restrictions. Generated with BioRender.

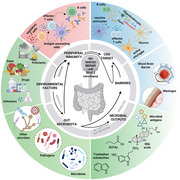

**Abbreviations**
ABC: adenosine triphosphate‐binding cassette
ADEM: acute disseminated encephalomyelitis
AHR: aryl hydrocarbon receptor
AIE: autoimmune encephalitis
AQP4: anti‐aquaporin 4
AMPAR: α‐amino‐3‐hydroxy‐5‐methyl‐4‐isoxazolepropionic acid receptor
BBB: blood‐brain barrier
CNPase2': 3'‐cyclic nucleotide 3'‐phosphodiesterase
CNS: central nervous system
CSF: cerebrospinal fluid
DAO: diamine oxidase
DCA: deoxycholic acid
EAE: experimental autoimmune encephalomyelitis
ETX: Epsilon toxin
GABAB_BBR: γ‐aminobutyric acid type B receptor
GF: germ‐free
HCMV: human cytomegalovirus
IgG: Immunoglobulin
iMSMS: International MS Microbiome Study
KEGG: Kyoto Encyclopedia of Genes and Genomes
KIR: killer cell immunoglobulin‐like receptors
LCA: lithocholic acid
LPS: lipopolysaccharide
MAG: myelin‐associated glycoprotein
MAIT: mucosa‐associated invariant T
MAL: myelin and lymphocyte
MBP: myelin basic protein
MOB: Pmyelin‐associated oligodendrocytic basic protein
MOG: myelin oligodendrocyte glycoprotein
MOGAD: myelin oligodendrocyte glycoprotein antibody–associated disease
MR: Mendelian randomization
MS: multiple sclerosis
NMDAR: N‐methyl‐D‐aspartate receptor
NMOSD: neuromyelitis optica spectrum disorder
NPSLE: autoimmune encephalitis, neuropsychiatric lupus
NR2: anti‐NMDAR
NT1: type 1 narcolepsyHCRT hypocretin
PBMCs: peripheral blood mononuclear cells
PLP: roteolipid protein
RASGRP2RAS: guanyl‐releasing protein 2
SCF: short‐chain fatty acids
SLE: systemic lupus erythematosus
TCC14: autoreactive T‐cell clone
Th: T helper
TLR: toll‐like receptor
Tregs: regulatory T cells

## 1 Introduction

Microbiota refers to a diverse collection of microorganisms from various kingdoms. This includes prokaryotes, such as bacteria and archaea, as well as eukaryotes, which encompass groups like protozoa and fungi. The composition and density of these microbial populations within the gastrointestinal tract, from the oral cavity to the rectum, are influenced by various factors. These include the host's pathophysiological characteristics, specific physical/biochemical parameters such as pH and oxygen levels, the presence of the mucosal layer, but also diet and drug consumption [1, 2, 3, 4]. Bacteria represent the most studied component of the human gut microbiota and the subject of significant research aimed at understanding their impact on health and disease. However, recent years have seen a growing emphasis on the role and composition of fungi, viruses, bacteriophages, and protozoa as researchers explore these entities both as potential therapeutic targets and as underlying causes of disease [5, 6, 7, 8].

Increasing evidence indicates that the microbiota plays a significant role in influencing both the onset and progression of different disease conditions, including autoimmune diseases, via metabolites, secreted products, or molecular mimicry affecting the immune system. Perturbation of the intestinal microbiota composition, a condition called dysbiosis, could lead to inflammation and activation of the immune system, which are linked to autoimmunity [9, 10].

This review will focus on the impact of the microbiota in central nervous system (CNS) autoimmune diseases via immune system involvement. Several clinical and experimental studies have established that the gut microbiota significantly influences immune system development from birth through weaning and into adulthood [11, 12, 13, 14, 15, 16, 17]. This extends to the modulation of both innate and adaptive immune responses, affecting mucosal tissues, secondary lymphoid and distal tissues and the systemic immunity, impacting the CNS by influencing microglial maturation, maintaining the integrity of the blood–brain barrier (BBB), and regulating the balance between effector and regulatory lymphocytes, thereby shaping susceptibility to autoimmune diseases and neuroinflammatory conditions [10, 18, 19, 20, 21]. In this review, we highlight the role of the human microbiota as a central environmental determinant of CNS autoimmunity through its impact on the host immune system, starting with evidence from patient studies and demonstrating functional relevance in animal models. Although various microbes, including fungi, viruses, and other infectious agents, have been implicated in the etiology of CNS autoimmune diseases, they are not the primary focus of this review, but are instead summarized in Table 1 [22, 23, 24, 25, 26, 27, 28, 29, 30, 31, 32, 33, 34, 35], where we also highlight the immune mechanisms linked to them. Finally, we discuss current challenges and methodological limitations in the field and consider future directions for microbiota‐based therapeutic strategies.

**TABLE 1:** Summary of microbial and infectious agents implicated in CNS autoimmune diseases.

Disease	Study/Model	Microbe/Microbial molecule	Methods	Host/Self‐antigen	Level of Evidence	Immune Mechanism	OUTCOME IN MODEL	REFERENCE
MS	Clinical Cohort MS (*n* = 80) vs. CTRL (*n* = 240)	*C. famata*, *C. albicans*, *C. parapsilosis*, *C. glabrata* (Abs and circulating Ags)	Indirect IF, ELISA, SLOT‐Blot Ag detection from serum	N/A	Human case‐control study, adjusted for age & sex; serological analysis	Systemic *Candida* exposure may drive or amplify autoimmune responses; higher odds ratios for *C. parapsilosis* (OR ∼7.3) and *C. glabrata* (OR ∼3.0) antigenemia	MS patients had significantly ↑ Abs to multiple *Candida* spp. (esp. *C. albicans* and *C. glabrata*), and ↑ prevalence of *C. parapsilosis* antigens; association independent of immunotherapy or disease duration	[22]
MS	Clinical Cohort MS (*n* = 135) vs. 100 HC (*n* = 100)	*C. albicans* intracellular proteinase A (Specific Enzyme Activity, SEA)	Culture → skin, nail, vagina; germ tube test; DEAE‐cellulose) and Con‐A chromatography; SDS‐PAGE & immunoblot; proteinase A activity and neural network	N/A	Clinical cross‐sectional study; MS subtypes (RR, PP, SP, PR); EDSS correlation	SEA ↑ in MS isolates vs controls; correlated with disease severity (r=0.65, p<0.001); PR subtype had highest SEA (∼12,700 µmol/min/mg); protein concentration inversely correlated with SEA	↑ fungal protease activity strongly associated with disability progression (EDSS); suggests *C. albicans* protease may exacerbate CNS autoimmunity	[23]
MS	Clinical Cohort MS (*n* = 27), HC (*n* = 21), (including 5 twin pairs)	Gut yeasts*: C. albicans, C. parapsilosis, S. cerevisiae, S. delbrueckii, P. membranifaciens*	Culture (2000 isolates), ITS1 sequencing, metagenomics, phenotypic assays, PBMC stimulation, IF of MS brain	Fungal riboflavin derivatives via MR1; innate cytokines (IL‐23)	Clinical cohorts + ex vivo PBMC assays + MS autopsy brain tissue	Fungal extracts activated MAIT cells (CD161^+^Va7.2^+^CD8^+^); IL‐23–mediated proliferation; production of IL‐17, GM‐CSF, TNFα, CCL20; infiltration of MAIT cells into active MS brain lesions	MS patients showed ↑ fungal richness, ↑ Candida & Saccharomyces isolates; MAIT cells activated and proliferated strongly; infiltrated CNS and produced proinflammatory cytokines, potentially amplifying demyelination	[24]
POMS	POMS (*n* = 18) vs. monophasic ADS (*n* = 13) vs. controls (*n* = 15)	*↑ C. albicans, C. jadinii, F. poae* (predicted markers); *↑ A. bisporus* (dietary origin)	Stool samples, ITS2 amplicon sequencing (Illumina MiSeq), OTU clustering, α‐ and β‐diversity, differential abundance (ALDEx2, LEfSe), BLAST+ species‐level assignment	N/A	Cross‐sectional pediatric cohort; 46 participants (Canada)	Altered mycobiota diversity; Basidiomycota enriched in POMS; *Candida* spp. known to activate innate immunity (IL‐23/Th17 axis, MAIT activation); *Fusarium* spp. produce mycotoxins affecting sphingolipid metabolism	POMS participants showed distinct β‐diversity from monoADS (*p* = 0.005); predictive markers (*C. albicans*, *C. jadinii*, *F. poae*) strongly associated with POMS; supports fungal dysbiosis as a potential early contributor to CNS autoimmunity	[25]
MS	MS (*n* = 24) HD (*n* = 24)	*EBV* (EBNA1)	ELISPOT, western blot, flow cytometry, ELISA, and purification of recombinant EBNA1, regression assay, T cell cross‐recognition, Luminex assay	Myelin antigens: MBP, PLP, MOG, CNPase (also tested proinsulin)	Direct ex vivo and in vitro functional assays in primary patient cells	Expansion of EBNA1‐specific CD4^+^ Th1 memory cells (cross‐react with myelin antigens) produces IFN‐γ and IL‐2 (polyfunctional phenotype) and positive for CXCR3^+^, CD27^+^, and CD28^+^	Cross‐reactive EBNA1‐specific T cells recognized myelin peptides more than the control antigen (proinsulin). IL‐2 production was distinctive. Myelin reactivity detected in 3.37% of EBNA1‐specific T cells in MS	[26]
MS	Human CSF & blood; EAE SJL/J mouse model	*EBV* EBNA1 (AA386–405) transcription factor	antibody expression, protein & peptide microarrays, crystallography, PhIP‐seq, proteomics	GlialCAM (AA370–389, intracellular domain, phosphorylated Ser376/377)	Human CSF (*n* = 9 MS), PBMC assays, structural biology, EAE immunization in SJL/J mice	Molecular mimicry: anti‐EBNA1 antibodies cross‐react with GlialCAM; affinity enhanced by GlialCAM phosphorylation; CD4^+^ & CD8^+^ T‐cell responses	EBNA1 immunization in mice exacerbated EAE (↑ CNS infiltration, demyelination, paresis); in MS patients, anti‐EBNA1 and anti‐GlialCAM antibodies found in ∼20%–25%	[27]
MS	US military cohort; >10 million people screened; MS cases (*n* = 955)	*EBV* infection (EBNA, VCA antibodies; EBNA1‐specific peptides enriched)	Prospective 20‐year cohort; > 62 million serum samples; EBV/CMV serology; VirScan virome‐wide screening; neurofilament light chain (sNfL) assay	EBNA1 most strongly associated	Human longitudinal cohort; unmatched sample size; pre‐onset and post‐onset serum analysis	EBV seroconversion precedes MS onset by ∼7.5 years; EBV antibodies ↑ before MS, not seen for CMV/other viruses; sNfL ↑ only after EBV infection → EBV is causal, not a bystander	Risk of MS ↑ 32‐fold after EBV infection; no association with CMV; virtually all MS cases EBV‐positive before onset; supports EBV as necessary cause	[28]
MS	Clinical Cohort, MS (*n* = 270) vs. matched controls (*n* = 270)	*EBV* EBNA1 peptide (AA386–405)	ELISA (EBNA1‐specific & cross‐reactive GlialCAM Abs), flow cytometry (B cells, CD4+, CD8+, NK subsets), co‐culture assays, EBV/HCMV genotyping, functional NK cytotoxicity assays	GlialCAM (AA370–389, CNS adhesion molecule)	Human cohort study + mechanistic *ex vivo* assays	Molecular mimicry (EBNA1–GlialCAM); failure of immune control due to ↓ NKG2C^+^ and NKG2D^+^ NK cells; immune evasion via EBV‐induced HLA‐E upregulation and IL‐27 secretion; impaired HLA‐E–restricted CD8^+^ T‐cell responses	High EBNA1‐specific and cross‐reactive GlialCAM responses in MS and EBNA^high^ controls; only MS patients lacked protective NK (NKG2C/NKG2D) and CD8^+^ T‐cell responses; EBV variants (LMP1 peptides) increased HLA‐E expression → potent immune evasion; combined host‐virus risk factors ↑ MS risk up to 260‐fold	[29]
NMO	NMO (*n* = 15) vs healthy controls (*n* = 9)	*C. perfringens* ABC transporter permease peptide (aa204–217: FIILPVSMVLISLV)	Homology search (BLAST), PBMC proliferation assays (CFSE, [^3^H]thymidine), recall assays, HLA‐blocking	Aquaporin‐4 (AQP4) p61–80 (core epitope p63–76: EKPLPVDMVLISLC)	Human *ex vivo* study (15 NMO vs 9 healthy controls); HLA restriction confirmed (HLA‐DRB10301, DRB30202)	Molecular mimicry: cross‐reactive CD4+ T cells; Th17 polarization (↑IL‐17, IL‐6), ↑CD40/CD80 on monocytes; impaired Treg expansion	AQP4‐specific T cells proliferated strongly in NMO patients; cross‐reactivity with *C. perfringens* ABC peptide demonstrated; provides mechanistic link between gut microbe and CNS autoimmunity	[30]
MS	BMS, RRMS untreated, RRMS IFN‐treated, RRMS relapse, PPMS; MS (*n* = 98) vs. HC (*n* = 120)	Microbiota shifts: *↓* Butyricicoccus (SCFA‐producer), *↓* Gemmiger*, ↑* Clostridium cluster IV & XVIII*, ↑* Sporobacter, ↑ Methanobrevibacter; higher prevalence of Enterotype B2 in active RRMS	16S rRNA V4 sequencing, amplicon‐based diversity (Illumina MiSeq), enterotype analysis, statistical modeling with covariates (stool consistency, BMI, age, sex)	N/A	Cross‐sectional human cohort, phenotypically stratified MS subgroups	↓ *Butyricicoccus* associated with lower SCFA → reduced Treg induction; Enterotype B2 enriched in RRMS‐I and RRMS‐R → proinflammatory milieu; taxa shifts (↑ *Clostridiales, Methanobrevibacter*) linked to dysregulated immunity	Active RRMS showed microbial profiles consistent with proinflammatory activity; PPMS and BMS retained higher richness; *Butyricicoccus* inversely correlated with disease severity (ARMSS, EDSS, patient‐reported disability)	[31]
MS	POMS (MS *n* = 20, controls *n* = 20)	Altered metabolic pathways: ↑ lipopolysaccharide biosynthesis, ↑ capsule polysaccharides, ↑ methanogenesis; ↓ peptidoglycan, ↓ starch & resistant fiber metabolism; ↓ pullulanase (degrades pullulan and starch	Shotgun metagenomics (Illumina NextSeq), functional profiling (HMP Unified Metabolic Analysis, MIMOSA2), CAZyme annotation (dbCAN2), diversity metrics, Wilcoxon & PERMANOVA tests	N/A	Human cohort, Canadian Pediatric Demyelinating Disease Network	Reduced resistant starch metabolism → ↓ SCFA → impaired Treg/anti‐inflammatory tone; ↑ LPS biosynthesis → potential proinflammatory priming; metabolic imbalance more pronounced in DMD‐exposed MS	Pediatric MS participants displayed ↑ LPS/capsule biosynthesis and ↓ fiber/starch metabolism, suggesting functional dysbiosis with proinflammatory bias	[32]
MS	In silico	Viral: HPV‐B19 capsid, AAV‐4 capsid, EBV EBNA1, HHV‐6 U24; Bacterial: *M. paratuberculosis* proteins, *C. perfringens* epsilon toxin, *L. reuteri* UvrA/EF‐G, *E*. aminopeptidase; Fungal: *Aspergillus, Penicillium* 6‐methylsalicylic acid synthase; Others: *B. fragilis* polysaccharide A enzymes (Wzy, WcfQ), bile salt hydrolase (BSH); *SARS‐CoV‐2* spike & main protease	BLAST sequence alignments, PRALINE structural alignment, 3D modeling (YASARA, MUSTANG), AlphaFold, IEDB epitope prediction	MBP (AA179–222, 246–256); MOG (AA68–76, YRSPFSRVV); IRF5; Glial motifs	In silico bioinformatics; cross‐referenced with prior MS patient antibody/T‐cell data	Predicted molecular mimicry between microbial/fungal proteins and MBP/MOG epitopes; alignment with MS‐risk HLA‐DRB1*15:01 binding motifs	Suggests multiple pathogens (viral, bacterial, fungal) and gut commensals may contribute to MS pathogenesis via structural and sequence mimicry; highlights conserved motifs beyond primary sequence	[33]
SLE	Clinical cohort + gnotobiotic mouse models	Commensal Ro60 orthologs (from *P. propionicum, C. amycolatum, A. massiliensis, B. thetaiotaomicron)*	16S rRNA sequencing, qPCR of orthologs, human SLE immunoprecipitation/WB, T cell clone assays, HLA‐DR3+ patient PBMCs, monocolonization with *B. theta*	Human Ro60 protein (B and T cell epitopes: aa169–190, aa316–335)	In vitro human (memory T cells, lupus sera) + in vivo (GF and NOD mice monocolonized with Ro60^+^ commensals)	CD4+ T cell cross‐reactivity (memory clones responsive to commensal Ro60 and hRo60 peptides); lupus sera immunoprecipitate commensal Ro60‐Y RNA RNPs; B cell/antibody cross‐reactivity	Human lupus sera recognize commensal Ro60; GF mice monocolonized with *B. theta* develop anti‐hRo60 IgG, T cell proliferation, glomerular immune complex deposition; disease exacerbated with TLR7 agonist (IMQ)	[34]
MS	Clinical cohort Taiwan (HZ *n* = 315,550 vs. CTRL946,650)	VZV, reactivation → herpes zoster	Nationwide health insurance claims database; ICD‐9 coding for HZ and MS; 1‐year follow‐up; Cox proportional hazards regression	N/A	Clinical epidemiology (nationwide population‐based cohort)	Possible VZV‐driven immune dysregulation → triggers or accelerates CNS autoimmunity	Risk of developing MS within 1 year after HZ infection increased 3.96‐fold (95% CI 2.22–7.07, *p* < 0.001) vs. CTRL	[35]

Abs, antibodies; Ags, antigens; MS, multiple sclerosis; POMS, pediatric‐onset multiple sclerosis; ADS, acquired demyelinating syndrome; monoADS, monophasic ADS; NMO, neuromyelitis optica; SLE, systemic lupus erythematosus; HC(s), healthy control(s); CTRL, control(s); HD, Healthy donor(s); CNS, central nervous system; CSF, cerebrospinal fluid; EAE, experimental autoimmune encephalomyelitis; SPF, specific pathogen‐free; GF, germ‐free; SJL/J, SJL/J mouse strain; NOD, nonobese diabetic (mouse); BMS, benign multiple sclerosis; RRMS, relapsing–remitting multiple sclerosis; PPMS, primary progressive multiple sclerosis; RR/PP/SP/PR, relapsing–remitting/primary progressive/secondary progressive/progressive‐relapsing (MS clinical course); EBV, Epstein–Barr virus; EBNA1, Epstein–Barr nuclear antigen 1; VCA, viral capsid antigen; HCMV/CMV, (Human) cytomegalovirus; HHV‐6, human herpesvirus 6; HPV‐B19, human parvovirus B19; AAV‐4, adeno‐associated virus 4; SARS‐CoV‐2, severe acute respiratory syndrome coronavirus 2; HZ, Herpes Zoster; U24, HHV‐6 U24 protein; MAIT, mucosal‐associated invariant T cells; MR1, MHC class I‐related molecule; NK, natural killer; PBMC(s), peripheral blood mononuclear cell(s); CD4^+^/CD8^+^, cluster of differentiation 4/8; CD161/CD27/CD28, T‐cell surface markers; IL‐2/IL‐17/IL‐23, interleukin‐2/‐17/‐23; IFN‐γ, interferon gamma; GM‐CSF, granulocyte‐macrophage colony‐stimulating factor; CXCR3, C‐X‐C chemokine receptor 3; CCL20, C‐C motif chemokine ligand 20; TLR7, toll‐like receptor 7; IMQ, imiquimod; AQP4, aquaporin‐4; MOG, myelin oligodendrocyte glycoprotein; PLP, proteolipid protein; OVA, ovalbumin; IgG, immunoglobulin G; hRo60, human Ro60/SSA antigen; EDSS, expanded disability status scale; ARMSS, age‐related multiple sclerosis severity score; BMI, body mass index; OR, odds ratio; CI, confidence interval; NfL/sNfL, (serum) neurofilament light chain; ELISA, enzyme‐linked immunosorbent assay; ELISPOT, enzyme‐linked ImmunoSpot; WB, western blot; SDS‐PAGE, sodium dodecyl sulfate–polyacrylamide gel electrophoresis; IF, immunofluorescence; qPCR, quantitative polymerase chain reaction; PhIP‐seq, Phage ImmunoPrecipitation sequencing; VirScan, phage‐display serology platform; 16S rRNA, 16S ribosomal RNA; ITS1/ITS2, internal transcribed spacer 1/2; OTU, operational taxonomic unit; BLAST, basic local alignment search tool; PERMANOVA, permutational multivariate analysis of variance; LEfSe, linear discriminant analysis effect size; ALDEx2, analysis of differential abundance (ALDEx) 2; HMP, human microbiome project; IEDB, Immune Epitope Database; ICD‐9, International Classification of Diseases (nineth Revision); CFSE, carboxyfluorescein succinimidyl ester; Luminex, multiplex bead‐based assay platform; SCFA, short‐chain fatty acids; BSH, bile salt hydrolase; CAZyme, carbohydrate‐active enzyme; dbCAN2, CAZyme annotation pipeline; SEA, specific enzyme activity; ABC, ATP‐binding cassette (transporter); YASARA/MUSTANG/PRALINE, software tools used for modeling/alignments; N/A, not available.

## 2 CNS Autoimmune Disorders

An autoimmune disease is defined as a disease characterized by an excessive or inappropriate immune response against self‐antigens with excessive chronic inflammation, tissue damage, and/or dysfunction caused by the activation of T and/or B lymphocytes with a prolonged expression of specific autoantibodies and/or autoreactive T lymphocytes [36].

Autoimmune disorders within the CNS are demyelinating diseases, which include multiple sclerosis (MS), neuromyelitis optica spectrum disorder (NMOSD), myelin oligodendrocyte glycoprotein antibody–associated disease (MOGAD), autoimmune encephalitis (AIE), neuropsychiatric lupus (NPSLE), and narcolepsy.

MS is a chronic autoimmune and neurodegenerative disease affecting almost 3 million people worldwide [37]. It involves neuroinflammation, lymphocyte infiltration, demyelination, and axonal loss, and it is characterized by ataxia, loss of coordination, hyperreflexia, spasticity, visual and sensory impairment, fatigue, and cognitive difficulties. Most of the people with MS have lesions in the brain or both brain and spinal cord; fewer have spinal cord–only lesions and 50% of them will die because of MS. It is characterized by an important T cell and humoral B cell response against wide antigens, such as self‐antigens [38, 39] (e.g. myelin proteins like myelin‐associated glycoprotein (MAG), myelin basic protein (MBP) [40], proteolipid protein (PLP), myelin oligodendrocyte glycoprotein (MOG) [41, 42, 43, 44], 2',3'‐cyclic nucleotide 3'‐phosphodiesterase (CNPase), myelin‐associated oligodendrocytic basic protein (MOBP), non‐myelin proteins (αB‐crystallin [HspB5], S100β, transaldolase‐H, contactin‐2/TAG‐1), and nonprotein antigens (glycolipids)) targeting neurons, astrocytes, oligodendrocytes or ubiquitous self‐antigens and against viral antigens suggesting that environmental factors, such as microbial agents, could be linked to MS pathogenesis [45, 46, 47]. Autoproliferative T cells have been identified in MS; they recognize an autoantigen expressed in brain neurons and B cells, RAS guanyl releasing protein 2 (RASGRP2). These cells exhibited a T helper (Th)‐1 phenotype and depend on B cells for their proliferation [46, 47, 48].

NMOSD is an inflammatory demyelinating disease primarily affecting the optic nerves, spinal cord, and brainstem. Anti–aquaporin 4 (AQP4, a membrane‐bound water channel expressed abundantly on astrocytes)‐immunoglobulin (IgG) is present in 70%–90% of people affected by NMOSD. Serum titers of anti‐AQP4 antibodies are 1000‐fold higher than in the cerebrospinal fluid (CSF) and usually disappear with disease progression, implying that B cell activation and the origin of the humoral immune response are outside the CNS [49, 50]. AQP4‐specific T cell response has also described in peripheral blood mononuclear cells (PBMCs) of people affected by NMO [51, 52, 53]

MOGAD is an inflammatory demyelinating disorder of the central nervous system, often preceded by nonspecific prodromal symptoms such as fever, malaise, cough, or runny nose. It is characterized by optic neuritis, transverse myelitis of the gray matter, acute disseminated encephalomyelitis (ADEM), and brain stem/cerebellar symptoms such as ataxia, facial palsy, diplopia, and vertigo. Its clinical course may be monophasic or relapsing with persistent MOG‐IgG positivity and high antibody titers associated with higher relapse risk [54]. In contrast, identifying autoreactive T cells in MOGAD has proven to be challenging, suggesting that they may exist at very low frequency or are hard to detect [55]. Recently, a study of 24 MOGAD patients found that peripheral blood CD4^+^ T‐cells responded to MOG p16–40 and MOG p181–205 peptides in patients compared with controls [56].

AIE is characterized by different psychiatric and neurological symptoms with reduced synaptic plasticity but little neuronal loss [57] and is linked to various antibodies primarily targeting synaptic receptors, such as the N‐methyl‐d‐aspartate receptor (NMDAR), the α‐amino‐3‐hydroxy‐5‐methyl‐4‐isoxazolepropionic acid receptor (AMPAR), and the γ‐aminobutyric acid type B receptor (GABAB_BBR). NMDAR encephalitis is the most common autoimmune encephalitis, caused by IgG autoantibodies against the GluN1 subunit of the NMDAR, whose presence is more reliable in the CSF than in the serum, suggesting a CNS‐compartmentalized immune response [56]. While T cells are implicated, the specificity of the autoreactive T cells is often not well‐characterized in AIE. Neuron‐reactive cytotoxic CD8^+^ regulatory T cells expressing killer cell immunoglobulin‐like receptors (KIR) have been implicated in AIE [58].

NPSLE is a manifestation of systemic lupus erythematosus (SLE) characterized by neurological or psychiatric symptoms without an identifiable cause. The symptoms span from mood disorders to seizures, with potentially both ischemic and neuroinflammatory causes [59]. In NPSLE, a specific subset of serum and CSF anti‐NMDAR (NR2) antibodies has been implicated in the neuroinflammatory process. Experimental evidence suggests that anti‐NMDAR antibodies may exacerbate but not initiate neuropsychiatric symptoms. For instance, in animal models, these antibodies only trigger symptoms when administered directly into the central nervous system or after the BBB is compromised. Similarly, elevated antibody levels are observed only after behavioral symptoms develop [60]. This supports the idea that a breach in the brain's protective barriers is likely necessary for the antibodies to exert their harmful effects. As for AIE, also for human NPSLE, there is very limited direct evidence of autoreactive T cells; IFNγ‐producing myelin‐specific CD8^+^ T cells were detected only in the circulation of patients with NPSLE with white matter lesions [61].

Narcolepsy is a rare, chronic neurological disorder marked by uncontrollable daytime sleepiness and cataplexy. It results from the selective loss of neurons in the lateral hypothalamus that produce hypocretin, a neuropeptide essential for regulating the sleep‐wake cycle. Research into autoantibodies in narcolepsy, particularly type 1 narcolepsy (NT1), has yielded mixed results. While some studies found elevated auto‐antibodies against proteins such as TRIB2, HCRTR2, and NRXN1, these findings are inconsistent. Many of these antigens are not unique to hypocretin (HCRT) neurons and have also been found in healthy individuals or patients with other sleep disorders [62]. This suggests that autoantibodies may play only a secondary role in narcolepsy. However, increasing evidence points to a central role for autoreactive T cells in narcolepsy pathogenesis. Both CD4^+^ and CD8^+^ T cells targeting HCRT and TRIB2 have been detected in the blood and CSF of NT1 patients; although they are rare, this supports their role in directly killing HCRT neurons [63].

## 3 Microbiota‐Driven Modulation of Immune Responses in CNS Autoimmunity

All of the CNS autoimmune diseases discussed above have, to varying degrees, been associated with alterations in the composition of the gut microbiota when stool from people affected by the diseases is compared with that of healthy individuals [64, 65, 66, 67, 68, 69, 70]. Commensal microbes are considered potential contributors to disease pathogenesis, yet findings across studies have often been inconsistent or even contradictory. Such discrepancies may reflect genetic variability among cohorts, differences in geography, diet, or other environmental exposures. Monozygotic twin studies have shown only a 20%–30% concordance rate for disease development, further highlighting the critical role of environmental factors, including the gut microbiota, in shaping disease susceptibility [71, 72]. The microbiota can interact with the host, particularly with the immune system, either through antigen presentation via molecular mimicry or through the production and release of microbial products and metabolites, such as short‐chain fatty acids (SCFAs), tryptophan metabolites, bile acid metabolites (BAs), polyamines, and aryl hydrocarbon receptor (AHR) ligands [73]. In the next paragraphs, we summarize current knowledge on how the human microbiota may influence the host immune system in individuals with CNS autoimmune diseases, with a focus on the molecular mechanisms identified to date (Table 2 [74, 75, 76, 77, 78, 79, 81, 87, 92]). We also aim to focus on studies in which a causal relationship between specific bacteria and CNS autoimmunity has been suggested.

**TABLE 2:** Summary of recent human‐mouse studies elucidating molecular mechanisms by which the microbiota influences CNS autoimmunity.

			Microbial status | Housing								
Mouse model	Sex	Number	SPF	GF	Ex‐GF	Disease	Treatment/Procedure	Microbial changes	Immunological changes	Pathological changes	Reference
RR SJL/J	N/A	12–26/group	X	X	X	MS	FMT of GF RR mice with fecal samples from MS‐affected vs. healthy co‐twins; anti–IL‐10 neutralization in colonized mice	MS‐twin microbiota ↑ in *A. muciniphila*; ↓ *Sutterella* spp	Splenocytes from MS‐colonized mice: ↓ IL‐10 & IL‐17 secretion upon anti‐CD3/CD28 stimulation; blocking IL‐10 → strong ↑ in EAE incidence	MS‐colonized mice = ↑ spontaneous EAE	[74]
RR SJL/J	♀	8–85/group		X	X	MS	Oral gavage with 100 µL of the ileal microbiota suspension from MS twins into RR mice (approx. 2 × 10^6^ bacterial cells) at 8 weeks of age	↑ *D. succinatiphilus, P. buccae* in MS twins. ↑ *E. tayi* and *Lachnoclostridium* in MS twin RR Mice (disease‐inducing agents)	↑ Anti‐MOG IgG1 ab in colonized RR mice vs. GF mice. ↑ % of IL‐17^+^in CD4^+^ T cells in the spleen of colonized RR mice	Colonization with MS‐derived ileal microbiota was followed by EAE development after 7–12 weeks	[87]
C57BL/6	N/A	6–8/group	X	X	X	MS	Antibiotic treatment (drinking water) → 1% Amphotericin B, 1 mg/mL ampicillin, 1 mg/mL neomycin, 1 mg/mL metronidazole, and 0.5 mg/mL vancomycin; Oral gavage by *A. calcoaceticus, A. muciniphila*, or *P*. *distasonis* in a solution of 1.5% sodium bicarbonate at 10^8^ CFU/mouse every 2–3 days for 2 weeks MOG_35_–_55_/CFA	↑ *Acinetobacter* and *Akkermansia* in MS samples. ↓ *P. distasonis* in MS patients	*A. calcoaceticu →* ↓ CD25^+^FoxP3^+^ Tregs among PBMC treated with and ↑ effector CD4+ T cell that differentiated into IFNγ‐producing Th1 cells *A*. *muciniphila* → ↑ Th1 Cells differentiation, ↑ IFNγ^+^ Th1 Cells *P*. *distasonis →* ↑ % of CD25^+^ T cells among the CD3^+^CD4^+^ T cell population. ↑ CD25^+^IL‐10^+^ cells including CD25^+^IL‐10^+^FoxP3^−^ Tr1 cells *Ex vivo* mono‐colonization *A. calcoaceticus* → ↑ T cells differentiation into the IFNγ^+^ Th1 in CLN *P. distasonis* → ↑ CD4^+^IL‐10^+^ T cells in MLN and SPL. Lack of IL‐10^+^ Treg induction in MLN from MS microbiota‐colonized mice	↑ EAE disease scores in mice colonized with microbiota from MS patients vs. mice colonized with microbiota from healthy patients or GF mice	[92]
C57BL/6N C57BL/6J Muc2^−/−^ Muc2^+/+^	N/A	3–23/group	X	X	X	MS	MOG_35_–_55_/CFA colonization with defined microbial consortia; FR vs FF diets; FMT	*Akkermansia genus* ↑ in Muc2^−/−^ mice. A*. muciniphila* in SM14 *A. muciniphila* levels ↑ in the SM14 group on an FF diet, with no EAE differences between FR and FF diets ↑ GABA in non‐EAE mice with *A. muciniphila*‐containing microbiota Removal of *Akkermansia* altered transcriptional activity of *R. intestinalis* and *Marvinbryantia*. ↑ *F. prausnitzii* in SM13 mice. W/o *A. muciniphila* → ↑ of three mucin glycan‐degrading Bacteroidota species in SM13 *E. coprostanoligenes, P. dorei*, and *E. bolteae* reliably predict individual EAE development, regardless of microbiota composition and recipient genotype	T‐cell polarization (MLN, CLP): correlates with group‐level disease severity SM14‐colonized: ↑ Th17/IFNγ^+^ infiltration in SC; Diet modulated microbiota composition but not directly predictive of severity	Muc2^−/−^ mice demonstrated ↓ susceptibility to EAE vs Muc2^+/+^ and CR mice, regardless of diet SM14: severe EAE phenotype (↑ clinical scores, demyelination). SM13: reduced severity. SM01 (only *Akkermansia*): intermediate EAE. ICI of *B. ovatus* enabled individual‐level prediction of disease course	[75]
C57BL/6J	♀	20/group	X			MS	MOG_35_–_55_/CFA Daily oral gavage with *A. muciniphila* (5 × 10^6^, 5 × 10^7^, 5 × 10^8^ CFU/mL, 10 mL/kg) for 3 weeks before immunization; control = water	*A. muciniphila* treatment significantly improved diversity and abundance, shown by ↑ observed species, ACE Chao 1, and Shannon indices, alongside a ↓ Simpson index ↓ *Firmicutes/Bacteroidetes* ratio (*A. muciniphila* vs. Naïve) *A. muciniphila*: genus level: ↓ *Desulfovibrionacea*e, *Lachnospiraceae*, and *Alistipes*; ↑ *Prevotellaceae* UCG‐001. LEfSe Analysis → ↑ *Paraprevotella*, *Ruminiclostridium*, *Roseburia*, and ↓ *Streptococcus* abundance	↑‐dose *A. muciniphila* administration significantly ↓ Th1/Th17 differentiation, shown by ↓ CD4^+^ GM‐CSF^+^ and CD4^+^ IL17A^+^ populations in peripheral LNs Altered permeability and tight junction protein expression in the ileum (occludin, claudin‐5, e‐cadherin, JAM‐A). ↓ Production of IL17A, IFN‐γ, and GM‐CSF in the SILP of *A. muciniphila*‐treated mice. ↑ secretion of CD4^+^CD25^+^FOXp3T cells in EAE mice EAE mice ↑ hippocampal levels of ASC, cleaved caspase‐1, IL‐18, NF‐κB, p‐NF‐κB, and NLRP3 vs. naïve mice Treatment ↓ levels of ASC, cleaved caspase‐1, IL‐18, NF‐κB, and p‐NF‐κB	High‐dose *A. muciniphila* → attenuated EAE clinical scores; ↓ demyelination and inflammatory infiltrates in SC EAE mice showed ↓ open‐arm entries and time vs. naïve (EPM). Treated mice showed ↓ total distance and higher discrimination ratio/index (NOR) Treated mice ↑ time in the center than EAE mice. EAE mice ↓ distance, speed, and center time vs. naïve (OFT). Treated mice had ↑ platform crossings than EAE. EAE mice showed ↓ crossings and speed vs. naïve (MWM)	[76]
HLA‐DR3.DQ8 double‐transgenic mice (MHC II ^−^/^−^	♂ & ♀	20/group	X		X	MS	Antibiotic treatment (drinking water) → vancomycin, neomycin, metronidazole, ampicillin for 3 weeks; PLP_91–110_/CFA; Treatment: oral gavage with *P. histicola*, *P. melaninogenica*, *C. sputigena*, *E. coli* (1 × 10^8^ starting day 7 postimmunization	qPCR indicated *P. histicola* colonization in the stomach and jejunum/ileum Key genera enriched in naïve *and P. histicola*–treated mice: *Prevotella*, *Lactobacillus*, *Sutterella* → ↓ in medium‐treated EAE mice, but restored with *P. histicola* administration.	*P. histicola* treatment: ↑ IL‐10 and TGF‐β levels. *P. melaninogenica*: ↑ IL‐10. EAE suppression by *P. histicola*. *P. histicola* ↓ pathogenic PLP_91_–_110_–specific T cell responses and shifts cytokine production toward an anti‐inflammatory profile ↓ total CD4^+^ T cells, ↓ IFN‐γ^+^ (Th1) CD4^+^ T cells, and ↓ IL‐17^+^ (Th17) CD4^+^ T cells in CNS. Splenocytes, MLNs, CLNs → CD4+CD25+ Tregs isolated from *P. histicola*‐challenged mice also showed ↑ suppression of PLP_91–110_ specific CD4+ T effector cells compared with the Tregs isolated from naive, medium‐treated, or *E. coli*‐treated mice *P. histicola* ↑ the frequency and anti‐inflammatory activity of tolerogenic APCs while lowering their antigen presentation capabilities	EAE suppression by *P. histicola*. Only 25% of *P. histicola*‐treated mice developed EAE vs. 100% in medium‐treated controls. Disease severity was markedly ↓ and disease onset was significantly delayed *P. histicola* dose *for* EAE suppression was 1 × 10^8^ CFU/mL. Effective EAE suppression requires viable *P. histicola*. Medium‐treated HLA‐DR3.DQ8 mice = inflammation and demyelination in the brain and SC. *P. histicola*‐treated mice showed ↓ CNS inflammation and demyelination compared with controls *P. histicola*–treated mice ↓ BBB permeability, measured by FITC–albumin leakage, vs medium‐only controls and restored gut permeability to pre‐EAE levels, indicating protection of intestinal barrier integrity	[77]
C57BL/6 2D2 TCR	♂ & ♀	3–9/group	X	X	X	MS	MOG_35_–_55_/CFA Antibiotic treatment (drinking water) → ampicillin 1 g/L, vancomycin 0.5 g/L, neomycin 1 g/L, and metronidazole 1 g/L gavaged with overnight cultures of *L. reuteri* or OTU0002 bacteria. To colonize SFB, germ‐free mice were orally gavaged with faeces from SFB‐monocolonized mice	OTU 0002 was nearly absent in the SI of ampicillin‐treated mice. OTU0002 = *A. stercoricanis*. DSM 13633 closest type strain. OTU0002 ↑ SI microbiota, in mucosal epithelial cells but not in the caecum or colon. The OTU0002 genome contains genes for colonization and biofilm formation. Shotgun sequencing identified mimicry peptides from *Lactobacillus*, including UvrA	Monocolonization with OTU0002 ↑ TH17 and Treg cells in mice. ↑ IL‐17A production and ↑ TH17 cells were observed in OTU0002‐colonized mice. ↑ genes related to TH17 cell activation and SAA enhanced gene expression in CD4+ cells, ↑ IL‐17A, and GM‐CSF levels *L. reuteri* and OTU0002 signals activate MOG‐specific T cells. Co‐colonization ↑ EAE symptoms and cytokine responses compared with monocolonization. MOG_35–55_ mice ↑ CD4+ T cells in SI, ↓ microbiota depletion	Mice orally treated with ampicillin were protected from SC demyelination and the infiltration of IFNγ^+^ and IL‐17A^+^ CD4^+^ T cells OTU0002 colonization in EAE‐induced mice led to more severe symptoms than in GF mice, while *L. reuteri* (OTU0001) had no significant effect Co‐colonization increased EAE symptoms and cytokine responses compared with monocolonization	[78]
C57BL/6	♀	16/group	X			NMOSD	Antibiotic treatment (drinking water) → amphotericin (1 mg/kg), vancomycin (50 mg/kg), neomycin (100 mg/kg), metronidazole (100 mg/kg), and ampicillin FMT	Gut Microbiome in NMOSD patients (acute phase) → ↑ α‐diversity in NMOSD patients; ↑ abundance of *Parabacteroides* in NMOSD patients. ↑ abundance of *P. distasonis*. ↑ relative abundances of in *S. vestibularis C. leptum, C. asparagiforme, R. hominis, A. muciniphila, L. fermentum, V. parvula, Oscillibacter sp. CAG 241, L. salivarius*, in NMOSD patients ↓ of tryptophan synthesis and degradation of arginine in NMOSD patients; ↑ of GABA production, propionic acid production, isovaleric acid, Glutamate metabolism, Nitric oxide synthesis, S‐adenosylmethionine (SAM) synthesis, Amino acid (AA) degradation pathways, and 17‐β‐estradiol degradation in NMOSD patients in the acute phase. ↓ relative abundances of *C. fastidiosus*, *Roseburia sp*. CAG 471, *V. tobetsuensis*, *P. bacterium* CAG 139, *G. formicilis*, *T. muris*, *R. bicirculans*, *L. lactis*, *F. plautii*, and *S. cristatus* in NMOSD patients in the remission phase. ↑ relative abundance of *C. aldenense* in NMOSD patients in the remission phase. ↓ of propionic acid production in NMOSD patients in the remission phase	NFMT mice: blood → ↑ of IL‐6, IL‐17A, IL‐23; ↓ IL‐10; splenic CD4^+^: ↑ Th17 cells, ↓ Foxp3^+^ Tregs	SC DEGs enriched: ↑ IL‐1 response, chemokine activity, IFN‐γ response, innate immunity, neutrophil chemotaxis, IFN‐γ pathways	[79]
C57BL/6 WT Thy1.1 Syn.Cre mT/mG Rpl22^tm1.1Psam^ OT‐I OT‐I.Thy1.1 OT‐I.mT/mG	♂ & ♀	4–5/group	X			MS	MOG_35_–_55_/CFA BBB disruption with 200 ng pertussis toxin, i.p. Cuprizone diet	N/A	↑ of different MHC class I antigen processing and presentation genes in cortical neurons and retina in EAE vs. Cuprizone mice ↑ MHC class I expression on GFP^+^ axons in Cuprizone mice OT‐I CD8^+^ T cells proliferate and ↑ LFA‐1 in deep CLNs and SPL when neurons express OVA. OVA detected in CD11c^+^ DCs and Lyve‐1^+^ stromal cells; CD11c^+^CD8α^+^ DCs present SIINFEKL–H2‐Kb in CLNs Accumulation of OT‐I CD8^+^ T cells in the brain only when both demyelination and neuronal OVA are present; OT‐I cells accumulate near GFP^+^ neurons/axons in the hippocampus, cortex, and corpus callosum Neuronal MHC class I and β2M expression is ↑ in MS patient brain tissue	Cuprizone and EAE produce ↑ demyelination, especially in the corpus callosum and associated tracts MS, neuronal cell bodies and axons in the cingulate cortex and thalamic tracts show elevated β2M and HLA‐A,B,C	[81]

Abbreviations: AhR, aryl hydrocarbon receptor; APC, antigen presenting cell; AQP4, aquaporin‐4; ASC, apoptosis‐associated speck‐like protein containing a CARDEG; BBB, blood–brain barrier; CFA, complete Freund's adjuvant; CFU/CFUs, colony‐forming unit(s) OTU, operational taxonomic unit; CLN, cervical lymph node; CLP, cecal lamina propria; DAO, diamine oxidase; DC, dendritic cell; DEG, differentially expressed gene; D‐Lac, D‐lactic acid; dsDNA, double‐stranded DNA; EAE, experimental autoimmune encephalomyelitis; EPM, elevated plus maze; ERV gp70, endogenous retrovirus glycoprotein 70; Ex‐GF, ex‐germ‐free (ex‐germ‐free colonized); FF, fiber‐free diet; FISH, fluorescence in situ hybridization; FITC‐dextran, fluorescein isothiocyanate–dextran assay (gut permeability test); FMT, fecal microbiota transplantation; FoxP3, Forkhead box P3 (Treg marker); FR, fiber‐rich diet; GABA, γ‐aminobutyric acid; GF, germ‐free; GM‐CSF, granulocyte‐macrophage colony‐stimulating factor; HCs, healthy controls; HLA, human leukocyte antigen; i.p, intraperitoneal; I3C, indole‐3‐carbinolvv; IBA1, ionized calcium‐binding adapter molecule 1; ICI, IgA coating index; IFN‐γ, interferon gamma; IL, interleukin (es. IL‐10, IL‐17A, IL‐23, IL‐6, IL‐12p40); IPA, indole‐3‐propionic acid; JAM‐A, junctional adhesion molecule A; LFA‐1, lymphocyte function‐associated antigen‐1; LP, lamina propria; LPS, lipopolysaccharide; MELFUS, microbubble‐enhanced focused ultrasound; MHC, major histocompatibility complex; MLN, mesenteric lymph node; MOG, myelin oligodendrocyte glycoprotein; MOG35–55, immunodominant peptide of myelin oligodendrocyte glycoprotein; MS, multiple sclerosis; MWM, Morris water maze; NF‐κB, nuclear factor kappa‐light‐chain‐enhancer of activated B cells; NLRP3, NOD‐like receptor family pyrin domain containing 3 (inflammasome); NMDAR, N‐methyl‐D‐aspartate receptor; NMO‐IgG, neuromyelitis optica immunoglobulin G; NMOSD, neuromyelitis optica spectrum disorder; NOR, novel object recognition; OFT, open field test; OVA, ovalbumin; PBMC, peripheral blood mononuclear cell; pDC, plasmacytoid dendritic cell; PLP, proteolipid protein; PLP91–110, immunodominant peptide of proteolipid protein; POMS, pediatric‐onset multiple sclerosis; qPCR, quantitative polymerase chain reaction; SAA, serum amyloid A; SC, spinal cord; SCFA, short‐chain fatty acids; SFB, segmented filamentous bacteria; SI, small intestine; SLE, systemic lupus erythematosus; SPF, specific pathogen‐free; SPN, spleen; Tfh, T follicular helper cell; Th1/Th17, T helper 1/T helper 17 cells; TNFR1, tumor necrosis factor receptor 1; Treg, regulatory T cells; WT, wild‐type; β2GPI, beta‐2 glycoprotein I.

### 3.1 Multiple Sclerosis

Several studies based on Mendelian randomization (MR) analyses have begun to investigate the causal role of the microbiome in MS pathogenesis. Several microbial strains have been associated with an increased risk of MS (e.g., *Actinobacteria* class, *Bifidobacteriaceae* family, and *Lactobacillus* genus) while others have been associated with a reduced risk of MS (e.g., *Prevotella* spp., *Lachnospiranaceae* genus, and *Negativibacillus* genus). The largest risk effect was associated with *Ruminococcus torques*, while the largest protective effect was with *Akkermansia muciniphila* [82]. Some bacteria (*Anaerofilum* id.2053, *Ruminococcus* id.11374, *Ruminococcaceae UCG003* id.11361, *Ruminiclostridium* id.11355, and *Anaerotruncus* id.2054) have been predicted to cause MS based on microbiome studies of the International MS Genetics Consortium (IMSGC) [83]. In a different study, 576 pairs of people with MS and genetically unrelated healthy household controls were recruited to investigate relationships between the microbiota and MS susceptibility, progression, and treatment [84]. The researchers observed specific microbial shifts in people with MS. These shifts included an increased relative abundance of *A. muciniphila*, *Ruthenibacterium lactatiformans*, *Hungatella hathewayi*, and *Eisenbergiella tayi*, as well as a decreased relative abundance of *Faecalibacterium prausnitzii* and *Blautia* species. Untreated people with MS showed overrepresentation of phytate degradation pathways and reductions in pyruvate‐producing carbohydrate metabolism pathways. Microbiome composition, function, and metabolite profiles also varied with disease‐modifying treatments, with interferon‐β activity potentially linked to the upregulation of SCFA transporters. People with untreated MS had distinct microbial networks compared with healthy controls, which supports the role of specific gut microbiome changes in MS risk, disease progression, and treatment response [84, 85]. Changes in commensal bacteria were also closely associated with changes in the mycobiota of subjects with RRMS compared with healthy controls, with an increased abundance of *Basidiomycota, Candida*, and *Epicoccum* genera and decreased abundance of *Ascomycota* and *Saccharomyces* [86]. This results in an increased fungal‐to‐bacterial ratio compared with healthy controls.

Importantly, studies conducted within the MS TWIN study in Germany identified potential MS drivers among the microbiota. Untreated MS twins exhibited an increased abundance of specific taxa, including *Akkermansia*. When this microbiota was transplanted into germ‐free (GF) mice that were genetically susceptible to experimental autoimmune encephalomyelitis (EAE), it induced a higher incidence of disease than the microbiota derived from healthy twins. The colonized mice displayed stable microbial profiles with notable differences, such as reduced levels of *Sutterella*, a taxon associated with immunoregulation. Furthermore, T cells from MS‐microbiota‐colonized mice produced less IL‐10. Neutralizing IL‐10 in healthy‐microbiota‐colonized mice increased disease incidence [74]. Transferring bacteria isolated from the ileal content of monozygotic twin pairs discordant for MS into GF mice that were genetically susceptible to EAE, the one from the MS‐affected twins induced MS‐like disease at higher rates than the microbiota of healthy co‐twins, strongly implicating *E. tayi* and *Lachnoclostridium* [87]. However, the molecular mechanisms behind these findings remain unknown.

One possible mechanism by which the microbiota could modulate immune cells is through bacterial products and antigens. Bacterial toxins can be some of these triggers for MS[89]. Epsilon toxin (ETX), a pore‐forming member of the aerolysin family, is produced by Clostridium perfringens as an inactive protoxin that is activated in the gut by host proteases. ETX binds to its receptor, the myelin and lymphocyte protein (MAL) [89], which is localized in lipid rafts and mediates toxin binding, oligomerization, and membrane insertion and is expressed by CNS endothelial cells. After binding to MAL, ETX increases blood–brain barrier (BBB) permeability. Recent evidence has shown increased abundance of ETX‐producing C. perfringens in the gut microbiome of MS patients [88]. If used in the active EAE mouse model as an adjuvant in the MOG‐immunization protocol, ETX induced multifocal inflammatory demyelination in mice, resembling MS, more closely than those induced by pertussis toxin, and ETX‐mediated upregulation of genes in CNS endothelial cells linked to protease, signal transduction, cytokines, transcription factors, and BBB disruption [88]. MAL is also expressed on human lymphocytes; however, the direct effect of ETX on autoreactive T cells in MS has not been addressed [90].

Different forms of molecular mimicry have been proposed between bacterial antigens and MS self‐antigens [91]. In vitro stimulation of PBMCs with extracts from the total bacterial communities from the stool of healthy donors or donors with MS showed that PBMCs from people with MS had a significantly lower ability to differentiate into CD25^+^FoxP3^+^ regulatory T cells (Tregs). To identify the bugs responsible for these effects, in vitro stimulation was conducted by exposing PBMCs from healthy donors to a suspension of different bacterial extracts under different stimulating conditions (e.g., Treg, Th1, and Th17). In vivo colonization experiments were also conducted using antibiotic‐treated or GF mice with the same bacteria: *Acinetobacter calcoaceticus*, *A. muciniphila*, or *Parabacteroides distasonis*. *A. muciniphila* showed no clear effect in mice, in contrast to the in vitro results, which showed an increase in Th1 cells. *A. calcoaceticus* inhibited FoxP3^+^ Treg differentiation and promoted IFNγ^+^ Th1 responses in vitro and in vivo*. P. distasonis* enhanced CD4^+^IL‐10^+^ T lymphocyte differentiation in vitro and in vivo, which is consistent with immunoregulatory activity. To assess the broader impact of MS‐associated microbiota, fecal samples from untreated people with MS and matched healthy controls were transplanted into GF mice, followed by EAE induction. Mice colonized with MS microbiota developed significantly more severe disease across all donor pairs tested and failed to generate IL‐10^+^ Tregs in mesenteric lymph nodes. RNA sequencing of spinal cords revealed the upregulation of immune response genes and the enrichment of microglia‐associated transcripts in mice colonized with MS microbiota [92]. In a different study, *Akkermansia* species were suggested to ameliorate MS. By isolating different strains of *Akkermansia* from healthy subjects, as well as from those with relapsing‐remitting (RR) and progressive MS, and then colonizing mice with them, researchers found that all of the strains lowered disease after immunization. This was accompanied by a reduction in RORγt^+^ and IL‐17‐producing γδ T cells. However, no effect was observed in FoxP3, RORγt, IL‐10, IFNγ, or IL‐17 expression in CD4^+^ or CD8^+^ T cells [93]. Despite these in vitro and in vivo evidence, the underlying mechanisms of T cell activation remain unclear. Although molecular mimicry may play a role, this has not been demonstrated, and alternative pathways, such as Toll‐like receptor (TLR) signaling, could also contribute or even predominate. In a different study, researchers analyzed peptides from *A. muciniphila*, which is overrepresented in MS patients. Using predictive peptide screening, they identified 30 *Akkermansia* peptides likely to stimulate an autoreactive T‐cell clone (TCC14) [94]. Nine of these peptides elicited strong T‐cell activation, comparable to that induced by the known self‐antigen RASGRP2. These responses involved typical activation markers (CD69 and CD25), cytokine production, and gene expression patterns reflecting Th2 differentiation and immune activation. In contrast, peptides from non‐MS‐associated microbes, such as human cytomegalovirus (HCMV) and *Prevotella histicola*, failed to stimulate T cells. Furthermore, some *Akkermansia* peptides also activated independent autoreactive T‐cell clones, inducing proliferation and IFN‐γ secretion [94]. Together, these findings indicate that the MS‐linked gut bacterium *A. muciniphila* harbors peptides that mimic self‐antigens, promoting activation and expansion of autoreactive CD4^+^ T cells, similarly to EBV, supporting cross‐molecular mimicry between bacterial and self‐antigens as environmental risk factors contributing to autoimmune activation in MS.

Additional studies investigated immunoglobulin (Ig)‐A levels in the gut of people with MS using bacterial flow cytometry and sequencing. Those with MS were found to have a higher proportion of IgA‐bound bacteria than the control group, including *A. muciniphila, Eggerthella lenta, Bifidobacterium adolescentis*, and certain *Ruminococcus* species. In contrast, *Bacteroides* taxa, which are associated with Treg responses, were found to be more IgA‐bound in healthy controls. Furthermore, during remission, people with MS tended toward higher levels of IgA binding to commensal bacteria compared with healthy controls, though this was not statistically significant. However, significantly fewer IgA‐bound gut bacteria were detected during acute relapse than during remission, suggesting that IgA‐producing cells may leave the gut during relapse. Further analysis revealed that CSF IgA levels increased relative to serum IgA levels during MS relapses, whereas IgG levels remained elevated regardless of disease state. This suggests intrathecal IgA production during relapses. Postmortem analysis of MS brain tissue revealed the presence of IgA^+^ plasma cells in meningeal, perivascular, and parenchymal lesions. Many of these cells expressed IL‐10 and gut‐homing markers, suggesting a gut origin. Furthermore, when CSF IgA was exposed to the gut bacteria of patients with active MS, it strongly bound to MS‐associated taxa (e.g., *A. muciniphila, E. lenta, F. prausnitzii*) but not to myelin or brain tissue. This confirms the reactivity of specific gut microbiota [95, 96]. Researchers have identified a correlation between increased intestinal Th17 cell frequency and a higher *Firmicutes/Bacteroidetes* ratio, as well as a higher relative abundance of *Streptococcus* and decreased *Prevotella* strains, in subjects with high disease activity RRMS, compared with healthy controls and MS patients with no disease activity [97]. Bacteria can modulate MS and affect the immune system by producing metabolites. People with RRMS have fewer gut bacteria that produce the necessary enzymes for secondary BA synthesis. These bacteria include *Bifidobacterium longum*, *Bifidobacterium pseudocatenulatum*, and *Christensenella minuta*, leading to lower levels of deoxycholic acid (DCA) in the intestine. BAs are synthesized as primary BAs in the liver and released in the upper intestine upon meal consumption, aiding fat digestion and regulating metabolism and immunity. Some primary BAs escape reabsorption and are deconjugated by gut bacteria and transformed into secondary BAs, such as DCA and lithocholic acid (LCA), and their derivatives. A reduction in intestinal DCA has been associated with an increased percentage of Th17 cells and a reduced frequency of mucosa‐associated invariant T (MAIT) cells in the peripheral blood of RRMS patients [98]. Additionally, they proved that soluble factors in RRMS fecal matter favor the differentiation of Th17 cells by inducing human naïve T cells from PBMCs toward a Th17 cell phenotype in vitro using RRMS fecal filtrate. Importantly, adding exogenous DCA to the in vitro culture reduced Th17 cell differentiation. The same result was achieved by supplementing DCA and LCA in a preclinical mouse model of MS, as this improved disease outcomes by boosting FoxP3^+^ Treg cells and suppressing Th17 cells in the gut, lymphoid tissues, and CNS [98].

Overall, these findings suggest that gut microbiota play a functional role in MS pathogenesis. However, the molecular basis of this phenomenon remains unclear.

### 3.2 Other CNS Autoimmune Diseases

Compared with MS, research on other CNS autoimmune diseases is still in the early stages, but growing interest in this field holds promise for uncovering new mechanistic insights in the future.

Metabolites of bacterial origin, such as SCFAs, have been found to be altered in the CSF and urine of people with NMOSD and MS [99, 100]. In various forms of optic neuritis, both with and without treatment, gut microbiota composition was altered, and changes in the abundance of specific bacteria, such as *Bacteroides* and *Escherichia*, correlated with immune mediators, *Bacteroides* negatively with IL‐21, IL‐17E, and TNF‐α, and *Escherichia* positively with MIP‐3α, suggesting a potential role of these microbes in modulating immune responses depending on disease status and treatment [101].

Molecular mimicry has been discussed in the etiology of NMOSD [30, 80]. The core determinant of AQP4 peptides, p63–76, showed 64% homology to a peptide from an adenosine triphosphate‐binding cassette (ABC) transporter in *C. perfringens*, which could be both a commensal and a pathogen in humans [30]. T cells from a few patients cross‐reacted with this bacterial peptide [30]. AQP4‐specific T cells displayed Th17 polarization, and monocytes from individuals with NMOSD produced higher levels of IL‐6, a Th17‐promoting cytokine, suggesting an involvement of both the adaptive and innate immune compartments [30]. Gut microbiome analyses in NMOSD revealed altered taxa, including increased *Enterobacteriaceae* and *C. perfringens* and decreased *Prevotella copri* [102]. Overall, these findings underscore the importance of AQP4‐specific Th17‐biased T‐cell responses in NMOSD and suggest a possible link to *Clostridium* species, which will need to be addressed further. While the mechanism by which C. perfringens may influence MS has not yet been established in NMOSD, it is plausible that the bacterium might similarly affect BBB integrity also in this disease.

In a study comparing the gut microbiota of 23 patients with anti‐NMDAR encephalitis and 24 matched healthy controls using 16S rDNA sequencing, no significant differences in overall bacterial diversity, operational taxonomic unit distribution, or genus‐level composition were identified. Moreover, patients with ovarian teratoma–associated NMDAR encephalitis showed microbiota profiles comparable to controls. Although minor alterations in certain genera (*Clostridium XVIII*, *Clostridium IV*, *Oscillibacter*, *Prevotella*, and *Blautia*) were observed during the acute disease stage in a few patients, these differences did not remain significant after multiple testing correction [103]. In a different study of 54 people newly diagnosed with anti‐NMDAR encephalitis, distinct microbiota profiles were observed across clinical subgroups. Subjects with psychiatric symptoms showed higher abundances of *Latrodectus indistinctus*, *C. perfringens*, and *Alistipes*, while *Mitsuokella*, *Lachnospira*, and *Veillonella* were enriched in those without psychiatric symptoms [68]. The epilepsy subgroup was characterized by increased levels of *Eubacteriaceae*, YRC22, and *Pseudoramibacter*. In addition, several taxa (4 orders, 2 classes, 5 families, 13 genera, and a species) differed significantly between tumor and nontumor subgroups. Beyond compositional shifts, people affected by anti‐NMDAR encephalitis exhibited markedly elevated levels of D‐lactate, diamine oxidase (DAO), and lipopolysaccharide (LPS) compared with healthy controls, suggesting a link between gut dysbiosis and systemic metabolic disturbances. These metabolites also showed specific microbial associations: DAO correlated positively with *Acinetobacter*, *Pseudoramibacter*, and *Veillonella*; LPS with *Clostridium* and *Pseudoramibacter*; and D‐lactate with *Streptococcus* and *Anaerotruncus* but negatively with *Dialister* and *Anaerostipes*. Functional predictions further revealed 68 Kyoto Encyclopedia of Genes and Genomes (KEGG) pathways that differed significantly between patients and healthy controls, including modules related to fructose and mannose metabolism, amino acid metabolism, and glutathione metabolism. Notably, LPS biosynthesis pathways were overrepresented in patients, consistent with elevated serum LPS levels and supporting a role for proinflammatory signaling in facilitating antibody access to the brain. Together, these findings suggest that gut microbiota alterations in anti‐NMDAR encephalitis extend beyond compositional changes to functional and metabolic pathways that may influence immune responses and disease pathogenesis. Fecal microbiota from subjects with anti‐NMDAR encephalitis induced hyperactivity and cognitive deficits in antibiotic‐treated recipient mice, while also selectively enhancing splenic and small intestinal proinflammatory Th17 cells without affecting Treg populations, suggesting that altered gut microbiota may contribute to both behavioral and immune features of the disease [68].

In narcolepsy, the gut microbiota may influence the disease through the production of SCFAs and urolithins, modulation of inflammatory responses, and interactions with lipid metabolism, although more detailed mechanistic studies are needed [104].

## 4 Conclusions

Together, these findings underscore the pivotal role of the gut microbiota‐immune axis in central nervous system (CNS) autoimmunity, establishing the microbiota as a promising therapeutic target. Although alterations in microbial composition are consistently reported across CNS autoimmune diseases, it is unclear whether these alterations are causal drivers of disease onset or secondary effects of ongoing pathology or treatment. Many studies remain descriptive, but important progress, particularly in MS, has begun to reveal mechanistic links between disease‐associated microbial communities and CNS autoimmunity. Moving forward, progress will depend on larger patient cohorts across multiple diseases, as well as longitudinal biobank studies to capture the temporal dynamics of disease development. Additionally, integrative approaches that combine metagenomics and metabolomics, identification of specific antigens, metabolites, or molecules, preclinical animal models, and that extend beyond bacteria to include other microbial kingdoms will be essential to understanding the functional contributions of the microbiota under different pathophysiological conditions, not only in MS, including before, during, and after treatment and during disease flares.

## Author Contributions

M.C. and F.R. conceived the idea and wrote the manuscript. M.C. generated the tables and the graphical abstract.

## Conflicts of Interest

The authors declare no conflicts of interest.

## Permission to Reproduce Material from Other Sources

N/A

## Data Availability

Data sharing is not applicable to this article as no datasets were generated or analyzed during the current study.
